# Supplementary material for: Discovery and application of insertion-deletion (INDEL) polymorphisms for QTL mapping of early life-history traits in Atlantic salmon
Source: BMC Genomics. 2010 Mar 8;11:156. doi: 10.1186/1471-2164-11-156 (PMC2838853; doi:10.1186/1471-2164-11-156)
Supplement: Additional file 2 — Information on developed 76 locus single-run INDEL panel in Atlantic salmon. Information on fluorescence labeling, primer concentrations, PCR pooling and links to alignments, INDEL motifs and GENESCAN (Burge and Karlin 1997) predictions of genes/exons are available in html format. [file 1471-2164-11-156-S2.ZIP › Additionalfile2/snpsummary3060.html]

```
Cluster 616 Contig 2

prev  Summary    Contig List  next
```

Size of Consensus sequence = 1143

Number of sequences = 19

Minimum redundancy = 6

Key

A gi|117852233|gb|EG924929.1|EG924929 EST\_ssal\_evf\_27594 ssalevf mixed\_tissue Salmo salar cDNA Salmo salar cDNA clone ssal\_evf\_536\_224\_fwd 3', mRNA sequence  
B gi|117524153|gb|EG855880.1|EG855880 EST\_ssal\_eve\_16485 ssaleve thyroid Salmo salar cDNA Salmo salar cDNA clone ssal\_eve\_522\_143\_rev 5', mRNA sequence  
C gi|117524044|gb|EG855771.1|EG855771 EST\_ssal\_eve\_16387 ssaleve thyroid Salmo salar cDNA Salmo salar cDNA clone ssal\_eve\_522\_093\_rev 5', mRNA sequence  
D gi|85027627|gb|DW556283.1|DW556283 EST\_ssal\_rgb2\_20702 rgb2 Salmo salar cDNA clone ssal\_rgb2\_533\_299\_fwd 3', mRNA sequence  
E gi|85029364|gb|DW558020.1|DW558020 EST\_ssal\_rgb2\_22439 rgb2 Salmo salar cDNA clone ssal\_rgb2\_536\_185\_fwd 3', mRNA sequence  
F gi|117463761|gb|EG795980.1|EG795980 EST\_ssal\_evd\_15703 ssalevd thymus Salmo salar cDNA Salmo salar cDNA clone ssal\_evd\_519\_370\_rev 5', mRNA sequence  
G gi|117494813|gb|EG827030.1|EG827030 EST\_ssal\_eve\_39620 ssaleve thyroid Salmo salar cDNA Salmo salar cDNA clone ssal\_eve\_553\_350\_rev 5', mRNA sequence  
H gi|117484974|gb|EG817191.1|EG817191 EST\_ssal\_evd\_19451 ssalevd thymus Salmo salar cDNA Salmo salar cDNA clone ssal\_evd\_525\_001\_fwd 3', mRNA sequence  
I gi|117565343|gb|EG886319.1|EG886319 EST\_ssal\_evf\_44587 ssalevf mixed\_tissue Salmo salar cDNA Salmo salar cDNA clone ssal\_evf\_559\_225\_rev 5', mRNA sequence  
J gi|117565344|gb|EG886320.1|EG886320 EST\_ssal\_evf\_44588 ssalevf mixed\_tissue Salmo salar cDNA Salmo salar cDNA clone ssal\_evf\_559\_225\_fwd 3', mRNA sequence  
K gi|117856126|gb|EG928822.1|EG928822 EST\_ssal\_evf\_31097 ssalevf mixed\_tissue Salmo salar cDNA Salmo salar cDNA clone ssal\_evf\_541\_109\_rev 5', mRNA sequence  
L gi|117852232|gb|EG924928.1|EG924928 EST\_ssal\_evf\_27593 ssalevf mixed\_tissue Salmo salar cDNA Salmo salar cDNA clone ssal\_evf\_536\_224\_rev 5', mRNA sequence  
M gi|117484975|gb|EG817192.1|EG817192 EST\_ssal\_evd\_19452 ssalevd thymus Salmo salar cDNA Salmo salar cDNA clone ssal\_evd\_525\_001\_rev 5', mRNA sequence  
N gi|117863827|gb|EG936523.1|EG936523 EST\_ssal\_evf\_20439 ssalevf mixed\_tissue Salmo salar cDNA Salmo salar cDNA clone ssal\_evf\_525\_338\_fwd 3', mRNA sequence  
O gi|117863828|gb|EG936524.1|EG936524 EST\_ssal\_evf\_20440 ssalevf mixed\_tissue Salmo salar cDNA Salmo salar cDNA clone ssal\_evf\_525\_338\_rev 5', mRNA sequence  
P gi|85026002|gb|DW554658.1|DW554658 EST\_ssal\_rgb2\_19077 rgb2 Salmo salar cDNA clone ssal\_rgb2\_531\_080\_fwd 3', mRNA sequence  
Q gi|117866216|gb|EG938912.1|EG938912 EST\_ssal\_evf\_22590 ssalevf mixed\_tissue Salmo salar cDNA Salmo salar cDNA clone ssal\_evf\_529\_286\_rev 5', mRNA sequence  
R gi|117856125|gb|EG928821.1|EG928821 EST\_ssal\_evf\_31096 ssalevf mixed\_tissue Salmo salar cDNA Salmo salar cDNA clone ssal\_evf\_541\_109\_fwd 3', mRNA sequence  
S gi|89831584|gb|DY693476.1|DY693476 EST\_ssal\_rgb2\_49215 ssalrgb2 mixed\_tissue Salmo salar cDNA Salmo salar cDNA clone ssal\_rgb2\_579\_292\_rev 5', mRNA sequence

2 SNPs detected

A B C D E F G H I J K L M N O P Q R S  cosegregation weighted

966 . . . . . - C - C C C C - - - - C C -   2/2 73.68
967 . . . . . - A - A A A A - - - - A A -   2/2 73.68
